# Dietary cardenolides enhance growth and change the direction of the fecundity‐longevity trade‐off in milkweed bugs (Heteroptera: Lygaeinae)

**DOI:** 10.1002/ece3.8402

**Published:** 2021-11-30

**Authors:** Prayan Pokharel, Anke Steppuhn, Georg Petschenka

**Affiliations:** ^1^ Department of Applied Entomology Institute of Phytomedicine University of Hohenheim Stuttgart Germany; ^2^ Department of Molecular Botany Institute of Biology University of Hohenheim Stuttgart Germany

**Keywords:** cardenolides, fitness costs, life history traits, milkweed bugs, Na^+^/K^+^‐ATPase, sequestration, trade‐off

## Abstract

Sequestration, that is, the accumulation of plant toxins into body tissues for defense, was predicted to incur physiological costs and may require resistance traits different from those of non‐sequestering insects. Alternatively, sequestering species could experience a cost in the absence of toxins due to selection on physiological homeostasis under permanent exposure of sequestered toxins in body tissues. Milkweed bugs (Heteroptera: Lygaeinae) sequester high amounts of plant‐derived cardenolides. Although being potent inhibitors of the ubiquitous animal enzyme Na^+^/K^+^‐ATPase, milkweed bugs can tolerate cardenolides by means of resistant Na^+^/K^+^‐ATPases. Both adaptations, resistance and sequestration, are ancestral traits of the Lygaeinae. Using four milkweed bug species (Heteroptera: Lygaeidae: Lygaeinae) and the related European firebug (Heteroptera: Pyrrhocoridae: *Pyrrhocoris apterus*) showing different combinations of the traits “cardenolide resistance” and “cardenolide sequestration,” we tested how the two traits affect larval growth upon exposure to dietary cardenolides in an artificial diet system. While cardenolides impaired the growth of *P*. *apterus* nymphs neither possessing a resistant Na^+^/K^+^‐ATPase nor sequestering cardenolides, growth was not affected in the non‐sequestering milkweed bug *Arocatus longiceps*, which possesses a resistant Na^+^/K^+^‐ATPase. Remarkably, cardenolides increased growth in the sequestering dietary specialists *Caenocoris nerii* and *Oncopeltus fasciatus* but not in the sequestering dietary generalist *Spilostethus pandurus*, which all possess a resistant Na^+^/K^+^‐ATPase. We furthermore assessed the effect of dietary cardenolides on additional life history parameters, including developmental speed, longevity of adults, and reproductive success in *O*. *fasciatus*. Unexpectedly, nymphs under cardenolide exposure developed substantially faster and lived longer as adults. However, fecundity of adults was reduced when maintained on cardenolide‐containing diet for their entire lifetime but not when adults were transferred to non‐toxic sunflower seeds. We speculate that the resistant Na^+^/K^+^‐ATPase of milkweed bugs is selected for working optimally in a “toxic environment,” that is, when sequestered cardenolides are stored in the body.

## Graphical abstract

We found that dietary cardenolides can increase growth, developmental speed, and longevity in milkweed bugs (Heteroptera: Lygaeinae).

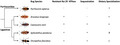

## 1 INTRODUCTION

Chemical defenses are widespread among animals and remarkably diverse across species. Many insect herbivores accumulate secondary metabolites from their host plants and utilize them for their defense to ward off natural enemies, a phenomenon called sequestration (Opitz & Müller, 2009; Petschenka & Agrawal, 2016). For example, caterpillars of the monarch butterfly (*Danaus plexippus*) sequester cardenolides from milkweed plants (*Asclepias* spp.). On the contrary, other insects produce their toxins via *de novo* synthesis as observed in leaf beetles (Chrysomelidae) (Pasteels et al., 1988) or butterflies producing cyanogenic glycosides (Heliconiinae) (Brown & Francini, 1990). Sequestration of toxins from plants and *de novo* synthesis of toxins may trade‐off evolutionarily as was suggested for *Heliconius* butterflies (Engler‐Chaouat & Gilbert, 2007) indicating a cost of both strategies. Under an ecological view, costs of possessing defenses are assumed to be compensated by gained protection against predators (Bowers, 1992; Camara, 1997).

It was speculated that the physiological costs of *de novo* synthesis of defensive compounds are higher compared to the costs of sequestration of plant toxins (Fürstenberg‐Hägg et al., 2014; Zvereva & Kozlov, 2016), which may explain why sequestration is a common phenomenon currently reported for more than 250 insect species acquiring toxins from at least 40 plant families (Opitz & Müller, 2009). Nevertheless, sequestration of chemical defenses may incur physiological costs (Camara, 1997; Reudler et al., 2015) since sequestering insects are exposed to high concentrations of toxins stored within their body tissues. In accordance, physiological costs of toxin resistance (here: insecticides) such as reduced energetic resources, lifespan, and fecundity have been shown in several insects including mosquitoes (Carriere et al., 1994; Rivero et al., 2011) and bed bugs (Gordon et al., 2015). Besides physiological costs, sequestration may interfere with the insect immune system as indicated by a compromised immune response of buckeye caterpillars (*Junonia coenia*) dependent on the amount of iridoid glycosides in their diet (Smilanich et al., 2009). Contrarily, dietary toxins were also suggested to interact positively with the immune system of sequestering insects such as caterpillars of the monarch butterfly (Tan et al., 2019) and the tobacco hornworm (*Manduca sexta*) (Garvey et al., 2021) indicating system‐specific differences. However, empirical evidence on the benefits of defenses is more apparent than their costs, and the costs of chemical defenses are not always easy to detect (Lindstedt et al., 2010; Ruxton, 2014). In line with this, evidence for actual physiological costs such as negative effects on growth or other fitness parameters like longevity and fecundity is very scarce (Zvereva & Kozlov, 2016) and understanding the costs of sequestration will require reductionist comparative analyses integrating a diversity of physiological and ecological parameters.

Trade‐offs play a crucial role in an organism's life history and occur when a beneficial change in one trait is linked to an unfavorable change in another trait causing a cost (Stearns, 1989). Life history theory suggests that fitness determining traits such as longevity and fecundity are negatively associated with each other (Flatt, 2011; Holliday, 1994). However, results are contradictory with studies showing positive, negative, or zero correlation between these two traits among individuals within a population (Bell, 1986; Van Noordwijk & de Jong, 1986). Generally, life history trade‐offs result from compromises in resource allocation across growth, survival, maintenance, and reproduction under challenges such as predation occurring in an ecosystem (Levins, 1968; Roff, 1992; Sibly & Calow, 1986; Walsh & Reznick, 2010). Regarding chemical defense, an organism's potential physiological costs may be estimated as trade‐offs between investments in defense and other physiological parameters such as growth, longevity, or fecundity (Camara, 1997; Ruxton et al., 2019).

Cardenolides are produced by more than 10 different plant families (Luckner & Wichtl, 2000; Malcolm, 1991) and are toxic to animals because they specifically inhibit the ubiquitous enzyme Na^+^/K^+^‐ATPase (Emery et al., 1998; Lingrel, 1992). Na^+^/K^+^‐ATPase is a cation carrier responsible for essential physiological functions such as the generation of neuronal action potentials and maintenance of an electrochemical gradient across the cell membrane (Jorgensen et al., 2003). Remarkably, insects from at least five orders, including milkweed bugs (Heteroptera: Lygaeinae), milkweed butterflies (Lepidoptera: Danaini), and leaf beetles (Coleoptera: Chrysomelidae), show common adaptations to cardenolides (Dobler et al., 2015). Resistance in these groups is mediated by target site insensitivity due to a few amino acid substitutions in the first extracellular loop of the alpha subunit of Na^+^/K^+^‐ATPase (ATPα), resulting in a high level of molecular convergence, that is, often the identical amino acid substitutions at the same positions confer resistance (Dobler et al., 2012, 2015; Zhen et al., 2012).

Milkweed bugs are seed feeders primarily found on Apocynaceae species and on unrelated cardenolide‐producing plants (Petschenka et al., 2020). Besides feeding on cardenolide‐containing plants, milkweed bugs also sequester cardenolides to ward off predators (Evans et al., 1986; Pokharel et al., 2020). Alteration of the Na^+^/K^+^‐ATPase in the Lygaeinae is probably correlated with several duplications of the ATPα1 gene resulting in four ATPα1 paralogs (A, B, C, and D) found in *Oncopeltus fasciatus* and *Lygaeus kalmii* (Dalla & Dobler, 2016; Yang et al., 2019; Zhen et al., 2012). Moreover, cardenolide‐resistant Na^+^/K^+^‐ATPases and the ability to sequester cardenolides are most likely synapomorphic traits of the Lygaeinae, which may account for the milkweed bugs' evolutionary success (Bramer et al., 2015).

**FIGURE 1:**
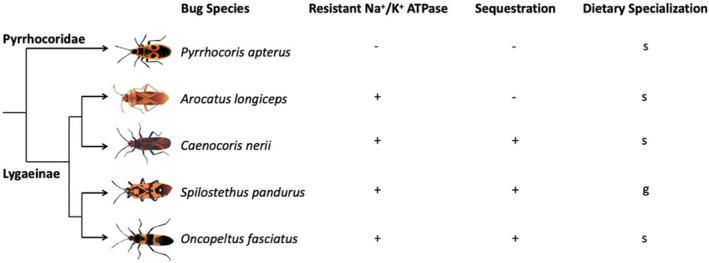
Overview of the Heteroptera species used in the experiments with their key traits. We compared four species of Lygaeinae sharing cardenolide‐resistant Na^+^/K^+^‐ATPase (+ = resistant; − = sensitive) but differing in their ability to sequester cardenolides (+ = sequestering; − = not sequestering). Within the sequestering milkweed bug species, *Oncopeltus fasciatus* and *Caenocoris nerii* may be classified as host‐plant specialists (s = specialist), while *Spilostethus pandurus* uses a wide variety of host plants (g = generalist). *Arocatus longiceps* is specialized on *Platanus* and *Ulmus* which are devoid of cardenolides, and lost its ability to sequester cardenolides in the course of evolution. *Pyrrhocoris apterus* belongs to the relatively closely related family Pyrrhocoridae and is specialized on cardenolide‐free Malvaceae. Furthermore, it is known to possess a sensitive Na^+^/K^+^‐ATPase, and, based on our recent analyses, does not sequester cardenolides. Phylogenetic relationships of Heteroptera species are based on Bramer et al. (2015)

The goal of our study was to evaluate if cardenolide exposure and sequestration causes physiological costs or benefits, and if these effects differ across closely related milkweed bug species possessing different combinations of the traits “resistance” and “sequestration” (i.e., having resistant/sensitive Na^+^/K^+^‐ATPases and sequestering/not sequestering). Our set of species included dietary specialist and generalist milkweed bug species as well as the European linden bug *Pyrrhocoris apterus* (Linnaeus, 1758, Pyrrhocoride) having no adaptations to cardenolides for comparison (Figure 1).

The milkweed bug species we used were *O*. *fasciatus* (Dallas, 1852), *Caenocoris nerii* (Germar, 1847) (both resistant and sequestering, dietary specialists), *Spilotethus pandurus* (Scopoli, 1763) (resistant and sequestering, dietary generalist), and *Arocatus longiceps* (Stal, 1872) (resistant and not sequestering, dietary specialist). As Stearns (1992) considers manipulating a single factor the most reliable and informative method to measure costs (or trade‐offs, if any), we manipulated cardenolide concentration in the diet as a single factor. For this purpose, we established an artificial diet approach and raised larvae on increasing doses of cardenolides in the diet. We assessed growth over the course of development. Furthermore, we determined the amount of sequestered cardenolides using liquid chromatography (HPLC‐DAD).

To test for effects on potential trade‐offs between life history parameters due to dietary cardenolides, we investigated the influence of dietary cardenolides on the longevity and fecundity in *O*. *fasciatus*. Trade‐offs have been measured in the field (Clutton‐Brock, 1982; Clutton‐Brock et al., 1983) and in the laboratory (Partridge & Farquhar, 1981), for example, by genotypic studies in *Drosophila melanogaster* (Rose & Charlesworth, 1981a, 1981b), and by phenotypic studies in *Daphnia pulex* and *Platyias patulus* (Bell, 1984a, 1984b). How different traits will interact depends on ecological factors (e.g. nutrition or predation) and the physiological state (e.g. developmental stage or fecundity) of an organism. Thus, trade‐offs can change across different environments in different species or even within the same species. Therefore, a life history trade‐off probably may only appear in a species under a particular set of conditions, such as stress (Reznick, 1985).

To explicitly investigate the influence of dietary toxins in milkweed bug species, we set out to test the following hypotheses: (i) insect species having different physiological traits (resistance and sequestration) and ecological strategies (generalist and specialist) will react differently to dietary toxins, (ii) sequestering species will experience costs, either in the presence or absence of toxins, and (iii) the fecundity‐longevity trade‐off will be altered by dietary toxins.

## 2 MATERIALS AND METHODS

### 2.1 Preparation of artificial diet

We followed Jones et al.'s method to prepare an artificial diet for *Oncopeltus fasciatus* (Jones et al., 1986) but used a modified approach to offer the diet to the bugs. Sunflower seeds (25 g), wheat germ (25 g), casein (25 g), sucrose (10 g), Wesson's salt (4 g), vitamins (Vanderzant Vitamin mix, 5 g), methyl 4‐hydroxybenzoate (1 g), sorbic acid (0.5 g), olive oil (7.5 g), and toxins (only for the treatment groups, not for controls) were blended in 200 ml of water until the mixture was homogenous. Agar (7.5 g) was boiled separately in 300 ml of water in a microwave. After 5 min, when the agar had slightly cooled down, the agar and the mixture were combined and blended again. The obtained paste was poured into plastic boxes and stored at 4°C for further use. For the feeding assays, an aliquot of diet was filled into the lid of a 2 ml Eppendorf tube and sealed with a piece of stretched parafilm to be used as an “artificial seed.” Using portions from the same initial bulk of the diet, we added increasing amounts of an equimolar mixture of crystalline ouabain and digitoxin (Sigma‐Aldrich, Taufkirchen, Germany) to prepare diets with 2 (“low”), 6 (“medium”), or 10 (“high”) mg cardenolide/g dry weight of diet. The initial diet without cardenolides added was used as a control. We used the polar ouabain and the relatively apolar digitoxin to mimic the condition that plants typically produce an array of cardenolides with a wide polarity spectrum. The concentration of cardenolides in the diet was chosen to be in the range of natural cardenolide concentrations observed in *Asclepias* seeds (Isman, 1977). For each batch of diet prepared, we verified the concentrations of cardenolides across all dietary treatments by using high‐performance liquid chromatography (HPLC, Section 2.2).

### 2.2 Quantification of cardenolides

To verify the amount of cardenolides in the artificial diet, cubes of diet (approx. 20–25 mg dry weight) were freeze dried, weighed, and added to a 2 ml screw‐cap tube containing approximately 900 mg of zirconia/silica beads (ø 2.3 mm, BioSpec Products, Inc., Bartlesville, OK, US). One ml HPLC‐grade methanol containing 0.01 mg/ml of oleandrin (PhytoLab GmbH & Co. KG, Vestenbergsgreuth, Germany) as an internal standard was added to the tube, and diet samples were homogenized for two cycles of 45 s at 6.5 m/s in a Fast Prep™ homogenizer (MP Biomedicals, LLC, Solon, OH, US). After centrifugation at 16,100 g for 3 min, supernatants were transferred into fresh tubes. Original samples were extracted two more times with 1 ml of pure methanol as described above. All the supernatants of a sample were combined, evaporated to dryness under a stream of nitrogen gas, and resuspended with 100 μl methanol by agitating tubes in the Fast Prep™ homogenizer without beads. Subsequently, samples were filtered into HPLC vials using Rotilabo ^®^ syringe filters (nylon, pore size: 0.45 μm, ø 13 mm, Carl Roth GmbH & Co. KG, Karlsruhe, Germany). Finally, 15 μl of the extract was injected into an Agilent 1100 series HPLC (Agilent Technologies, Santa Clara, US) equipped with a photodiode array detector, and compounds were separated on an EC 150/4.6NUCLEODUR^®^ C18 Gravity column (3 µm, 150 mm × 4.6 mm, Macherey‐Nagel, Düren, Germany). Cardenolides were eluted at a constant flow rate of 0.7 ml/min at 30°C using the following acetonitrile–water gradient: 0–2 min 10% acetonitrile, 13 min 95% acetonitrile, 18 min 95% acetonitrile, 23 min 10% acetonitrile, and 5 min reconditioning at 10% acetonitrile. The same HPLC method was used for quantification of sequestered cardenolides in *O*. *fasciatus* and *P*. *apterus*, respectively. For the analysis of sequestered cardenolides in *C*. *nerii*, *S*. *pandurus*, and *A*. *longiceps*, we used a different acetonitrile–water gradient to achieve improved separation of polar cardenolides: 0–2 min 16% acetonitrile, 25 min 70% acetonitrile, 30 min 95% acetonitrile, 35 min 95% acetonitrile, 37 min 16% acetonitrile, and 10 min reconditioning at 16% acetonitrile. We interpreted peaks with symmetrical absorption maxima between 216 and 222 nm as cardenolides (Malcolm & Zalucki, 1996) and integrated peaks at 218 nm using the Agilent ChemStation software (B.04.03). The amount of cardenolides in a sample was quantified based on the peak area of the known concentration of the internal standard oleandrin.

### 2.3 Insect colonies

*Oncopeltus fasciatus* were obtained from a long‐term laboratory colony (originally from the United States) maintained on sunflower seeds. We collected specimens of *P*. *apterus* in the vicinity of linden trees (*Tilia* spp., Malvaceae) and specimens of *A*. *longiceps* from under the bark of plane trees (*Platanus* spp., Platanaceae) in Giessen, Germany. Specimens of *S*. *pandurus* and *C*. *nerii* were collected from a *Nerium oleander* habitat close to Francavilla di Sicilia, Messina, Sicily, Italy. In the laboratory, insect colonies were reared in plastic boxes (19 × 19 × 19 cm) covered with gauze in a climate chamber (Fitotron^®^ SGC 120, Weiss Technik, Loughborough, UK) at 27°C, 60% humidity, and a day/night cycle of 16/8 h under artificial light. We reared all insects on organic sunflower seeds (Alnatura GmbH, Darmstadt, Germany), supplied water in cotton‐plugged Eppendorf tubes, and included a piece of cotton wool as a substrate for oviposition. In addition to sunflower seeds, *P*. *apterus* was provided with approximately 10 freshly chopped mealworms twice a week.

For the experiments described below, we used first‐generation offspring from field‐collected *P*. *apterus* and *A*. *longiceps* (maintained as described above), whereas *S*. *pandurus* and *C*. *nerii* offspring were obtained from colonies maintained in the laboratory for more than four generations.

### 2.4 Growth assay

We carried out feeding assays to investigate the influence of increasing doses of dietary toxins on the growth of larvae of four species of milkweed bugs (*O*. *fasciatus*, *C*. *nerii*, *S*. *pandurus*, and *A*. *longiceps*) and an outgroup, *P*. *apterus*. These species either lack the ability to tolerate and sequester cardenolides (*P*. *apterus*), possess a cardenolide‐resistant Na^+^/K^+^‐ATPase, and can sequester cardenolides (*O*. *fasciatus*, *C*. *nerii*, and *S*. *pandurus*) or possess a cardenolide‐resistant Na^+^/K^+^‐ATPase but lost the ability to sequester (*A*. *longiceps*) (Figure 1). We placed three second instar (L2) larvae from the stock colonies in a Petri dish (60 mm × 15 mm, with vents, Greiner Bio‐One, Frickenhausen, Germany) lined with filter paper (Rotilabo^®^ round filters, Carl Roth GmbH & Co. KG, Karlsruhe, Germany) that was supplied with an artificial seed (either being devoid of cardenolides, or possessing a cardenolide concentration of 2, 6, or 10 µg/mg dry weight) and a water source (a 0.5 ml Eppendorf tube plugged with cotton wool). The artificial seeds were replaced once in 2 weeks. All Petri dishes were spatially randomized and maintained in a controlled environment (KBWF 240 climate chamber, Binder, Tuttlingen, Germany) at 21°C, 60% humidity, and a day/night cycle of 16/8 h under artificial light. The growth of larvae was assessed twice a week over a period of 3 weeks by sedating all bugs of a Petri dish with CO_2_ and weighing them jointly. After reaching adulthood, at least one bug per Petri dish was transferred to a toxin‐free diet for 10 days to avoid a potential bias from toxins remaining in the gut (i.e., not being sequestered) by purging. Finally, bugs were killed by freezing, freeze‐dried, weighed, extracted and analyzed by HPLC to estimate the amount of sequestered cardenolides (Section 2.2). We also estimated the amount of excretion products on filter paper that may provide an indication of food intake (Appendix S1). For *O*. *fasciatus*, growth assays were carried out in batches. Altogether three experiments (*n* = 10 per treatment for experiments I and II; *n* = 5 per treatment for experiment III) were carried out. Additionally, we also carried out growth assays using a different *O*. *fasciatus* strain (Figure S3). Sequestration of cardenolides in *O*. *fasciatus* was only evaluated in specimens from the experiments I and II.

### 2.5 Life history assays with *Oncopeltus fasciatus*

#### 2.5.1 Developmental time

Since the effects of dietary cardenolides on growth were most pronounced in *O*. *fasciatus*, we carried out a separate experiment to assess additional life history parameters including duration of larval development, adult lifespan, and body size under the influence of dietary cardenolides in this species. We chose the medium‐dose cardenolide (6 µg/mg dry weight) because we observed that *O*. *fasciatus* showed maximal growth on this diet in our previous experiment. The experimental setup was similar to that of the growth assay. However, here only one L2 larva was placed in each Petri dish to monitor the time of larval development. Petri dishes lined with filter paper either containing medium‐dose diet or control diet without toxins and a water source (Section 2.4) were spatially randomized and kept in a climate chamber (Fitotron^®^ SGC 120, Weiss Technik, Loughborough, UK) at 27°C, 60% humidity, and a day/night cycle of 16/8 h under artificial light. We checked the Petri dishes every day for dead individuals, raised the bugs until adulthood, and observed them until they died. We also measured the body length of adult males and females raised on the two different diets using a Vernier caliper.

#### 2.5.2 Reproductive fitness

We conducted two additional experiments to assess the effect of dietary cardenolides on reproductive fitness. We raised L2 larvae in bulk (around 50 individuals) until adulthood in plastic boxes (19 × 19 × 19 cm) covered with gauze either on two artificial seeds of medium‐dose or control diet and a water source (four Eppendorf tubes of 2 ml plugged with cotton wool). Boxes were kept in a climate chamber (Fitotron^®^ SGC 120, Weiss Technik, Loughborough, UK) at 27°C, 60% humidity, and a day/night cycle of 16/8 h under artificial light. Artificial seeds were replaced once in 2 weeks. At least 3 (but not older than 6)‐day‐old males and females from the same treatment were paired in Petri dishes (9 cm × 1.5 cm, with vents, Greiner Bio‐One, Frickenhausen, Germany) lined with filter paper and a water source (a 2 ml Eppendorf tube plugged with cotton wool). Additionally, we included a piece of cotton wool as a substrate for oviposition. Petri dishes were spatially randomized and kept under the same conditions as described above. In a first experiment, adult bugs were supplied with the same type of artificial diet that they were raised upon (i.e., either control or medium‐dose cardenolide artificial seeds). In nature, adults of *O*. *fasciatus* disperse after reaching adulthood and forage for other seeds besides *Asclepias* spp. (Feir, 1974). Therefore, we carried out a second experiment under the same conditions as described above in which pairs of bugs were supplied with approx. 20 sunflower seeds instead of artificial seeds. Since a substantial portion of eggs in both experiments were unviable (possibly due to the use of an artificial diet), we counted only hatchlings and not the total number of eggs produced by each female over its entire lifespan. Especially after transfer to sunflower seeds, viable eggs were only produced by 52% of the females (13 of 25) raised on the artificial diet without toxins and by only 39% of the females (10 of 26) raised on the artificial diet with toxins. Remarkably, the proportion of females laying viable eggs was much higher in females remaining on artificial diet (>80%; 13 of 15 females on the control vs. 12 of 15 females on the toxic diet). For statistical analysis, females producing no viable eggs were excluded and their inclusion did not change the direction of the results.

### 2.6 Statistical analysis

Statistical analyses were computed using JMP^®^ Pro 15 statistical software (SAS Institute, Cary, NC, US). All data were 1og_10_ transformed to achieve homogeneity of variances and normality of residuals. For feeding experiments, we analyzed sequential data on larval masses with repeated measures ANOVA followed by LSMeans Tukey HSD test to assess potential differences across treatments. We compared the amounts of sequestered cardenolides across treatments and milkweed bug species by ANOVA followed by LSMeans Tukey HSD test and included bug species and treatment as model effects. Additionally, we estimated Pearson's correlation coefficients between body mass and concentration of sequestered cardenolides in *O*. *fasciatus*. Body length data were analyzed by ANOVA followed by LSMeans Tukey HSD test, including sex, treatment, and the interaction between sex and treatment as model effects. Lifespan and number of hatchlings were analyzed by ANOVA followed by LSMeans differences Student's *t*‐test, including treatment as the model effect. For *O*. *fasciatus*, “experiment” was always included as a model effect in our statistical analysis. Sample sizes for every experiment are mentioned in the figure legends. Probability values <0.05 were considered statistically significant.

## 3 RESULTS

### 3.1 Influence of dietary cardenolides on growth

**FIGURE 2:**
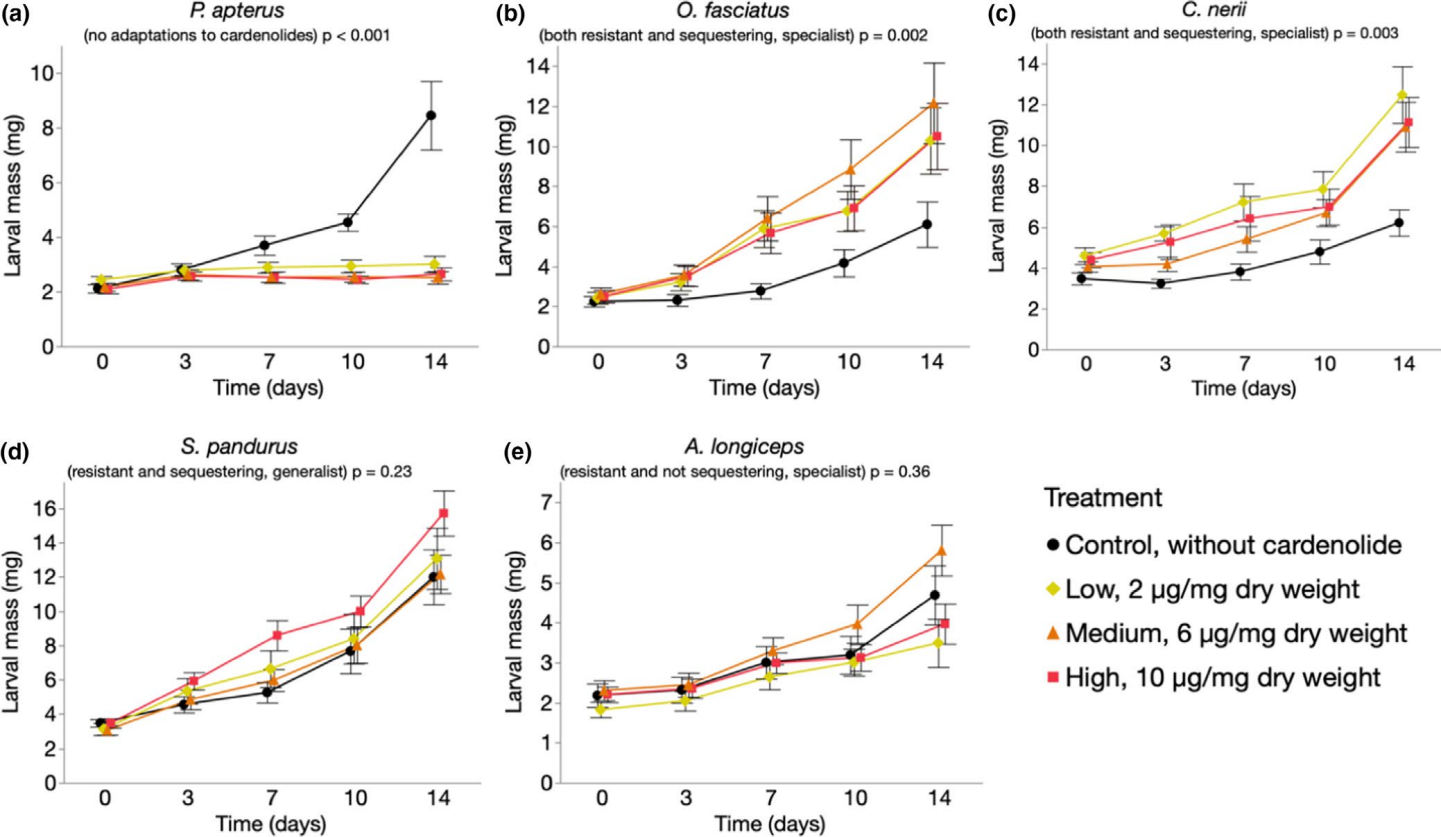
Growth of bugs on artificial diet with increasing doses of cardenolides. Each data point represents the mean (±SE) of larval mass at a given time. (a) *Pyrrhocoris apterus* (*n* = 10 per treatment), (b) *Oncopeltus fasciatus* (*n* = 25 per treatment, three experiments), (c) *Caenocoris nerii* (*n* = 10 per treatment), (d) *Spilostethus pandurus* (*n* = 10 per treatment), and (e) *Arocatus longiceps* (*n* = 10–13 per treatment)

We examined the influence of dietary toxins on the growth of *P*. *apterus*, *O*. *fasciatus*, *C*. *nerii*, *S*. *pandurus*, and *A*. *longiceps* (Figure 2) using an artificial diet containing increasing doses of cardenolides (Figure S1). Growth of *P*. *apterus* was compromised substantially [*F*(3, 36) = 8.83, *p* < .001] upon exposure to dietary cardenolides (*p* < .001, LSMeans Tukey HSD). In contrast, *S*. *pandurus* [*F*(3, 35.81) = 1.5, *p* = .23] and *A*. *longiceps* [*F*(3, 40) = 1.11, *p* = .36] grew equally well across all diets. Remarkably, cardenolides had a positive effect on growth in *O*. *fasciatus* [*F*(3, 88.01) * 5.33, *p* = .002] and *C*. *nerii* [*F*(3, 36) = 5.69, *p* = .003]. We observed increased growth in the presence of dietary toxins across all doses in *O*. *fasciatus* (low vs. control, *p* = .008; medium vs. control, *p* = .005; high vs. control, *p* = .015, LSMeans Tukey HSD). Compared to a diet without cardenolides, *C*. *nerii* grew better on the low‐ (*p* = .002) and the high‐dose (*p* = .02), but not on the medium‐dose diet (*p* = .09). Since our laboratory strain of *O*. *fasciatus* was highly inbred, we carried out the same experiment with a different laboratory strain of *O*. *fasciatus* and obtained similar results, but here only low‐dose was statistically significant from control (Figure S3).

The amount of excretion products was not influenced by the presence of toxins across diets for the milkweed bug species, *C*. *nerii* [*F*(3, 14) = 1.38, *p* = .29], *S*. *pandurus* [*F*(3, 15) = 0.53, *p* = .67], and *A*. *longiceps* [*F*(3, 5) = 0.96, *p* = .48], but there was an effect for *P*. *apterus* (lower in the presence of cardenolides, *F*(3, 15) = 4.69, *p* = .02). *Oncopeltus fasciatus* [*F*(3, 19) = 8.34, *p* < .001] excreted similar amounts when fed on either control, low‐, or medium‐dose diet, but less on the high‐dose diet compared to low (*p* = .004) and medium dose (*p* = .002), but similar to control (*p* = .25). This suggests that stronger growth in *O*. *fasciatus* and *C*. *nerii* is not due to increased food uptake mediated by a phagostimulatory effect of dietary cardenolides (Figure S4).

### 3.2 Cardenolide sequestration

**FIGURE 3:**
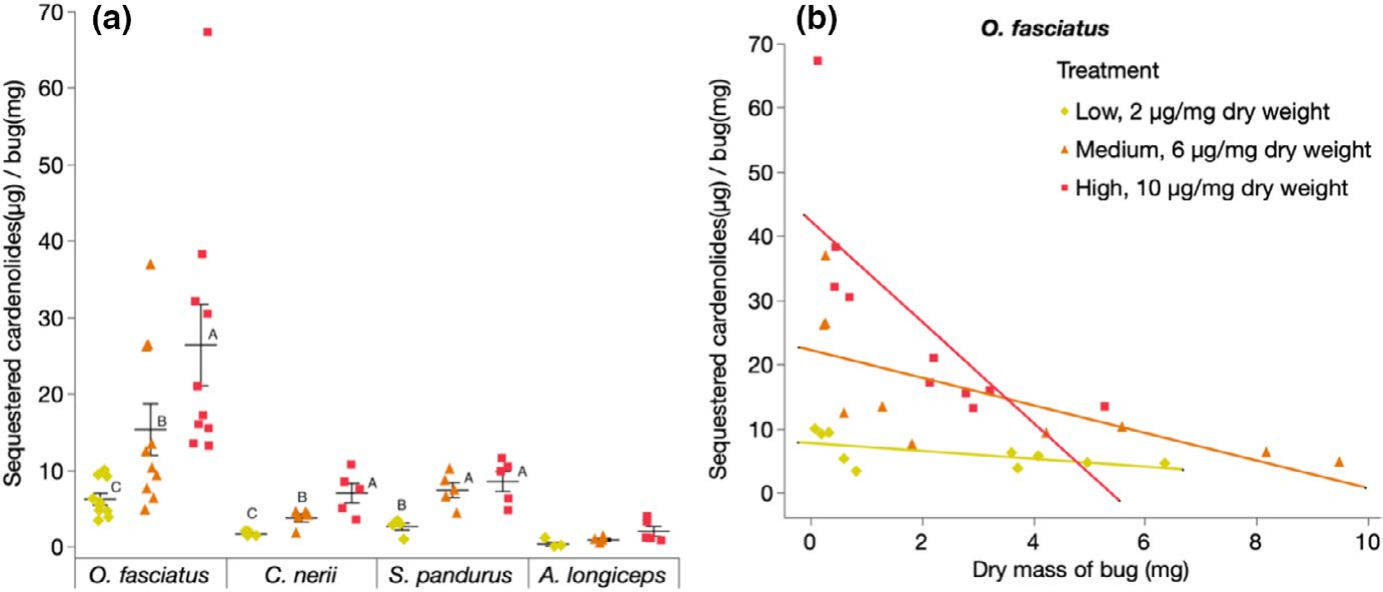
Sequestration of cardenolides by four species of milkweed bugs on artificial diet with increasing amounts of cardenolides. (a) Total amounts of cardenolides sequestered per species [*n* = 5, per treatment for all species except *Oncopeltus fasciatus* (*n* = 10)] and dietary cardenolide concentration. Horizontal bars represent the mean concentration (± SE) of sequestered cardenolides. Within the same bug species, different letters indicate significant differences across treatments and dots represent jittered raw data. (b) Correlations between dry body mass and concentration of sequestered cardenolides in *O*. *fasciatus* (*n* = 10 per treatment). Trend lines represent the linear least squares regression fits to data points and dots represent jittered raw data

*Pyrrhocoris apterus* did not sequester any cardenolides (Figure 3a). We found substantial differences regarding the concentration of sequestered cardenolides across all dietary treatments for *S*. *pandurus* [*F*(2, 12) = 12.11, *p* < .001], *O*. *fasciatus* [*F*(2, 27) = 15.99, *p* < .001], and *C*. *nerii* [*F*(2, 12) = 18.16, *p* < .001]. Compared to the other species, *O*. *fasciatus* sequestered remarkably higher amounts of cardenolides (*p* < .001). Although *A*. *longiceps* possesses a resistant Na^+^/K^+^‐ATPase, we observed only very small concentrations of sequestered cardenolides which is consistent with earlier findings (Bramer et al., 2015).

*Spilostethus pandurus* sequestered similar amounts of cardenolides from the diet with the intermediate and with the highest concentration of cardenolides (*p* = .89). In contrast, we found dose‐dependent cardenolide sequestration in *O*. *fasciatus* (low vs. medium, *p* = .011; low vs. high, *p* < .001; medium vs. high, *p* = .05) and in *C*. *nerii* (low vs. medium, *p* = .01; low vs. high, *p* < .001; medium vs. high, *p* = .05). Specifically, *O*. *fasciatus* sequestered 6.23 ± 0.76, 15.36 ± 3.39, and 26.43 ± 5.32; *C*. *nerii* sequestered 1.66 ± 0.14, 3.77 ± 0.52, and 7.02 ± 1.3 µg; and *S*. *pandurus* sequestered 2.64 ± 0.45, 7.43 ± 0.98, and 8.55 ± 1.29 µg cardenolides per mg dry weight (mean ± SE) from the low‐, medium‐, and high‐dose diet, respectively. Additionally, the sequestration data in *O*. *fasciatus* revealed an inverse relationship between body mass and concentration of sequestered cardenolides [low, *r*(10) = −0.7, *p* = .03; medium, *r*(10) = −0.92, *p* < .001; high, *r*(10) = −0.98, *p* < .001] (Figure 3b).

Besides the total amounts of cardenolides sequestered, we also compared the number of structurally different cardenolides across the sequestering species (Figure S2). Based on retention time comparison using an authentic standard, ouabain was sequestered as such. However, a peak with the retention time of digitoxin was not detected, but we found up to three compounds with a cardenolide spectrum and increased polarity probably representing digitoxin metabolites. Remarkably, we found more than one and up to three putative digitoxin metabolites in *S*. *pandurus* and *C*. *nerii*. In *O*. *fasciatus*, we did not find any digitoxin metabolites.

### 3.3 Influence of dietary cardenolides on lifespan, longevity, and body size of *Oncopeltus fasciatus*

**FIGURE 4:**
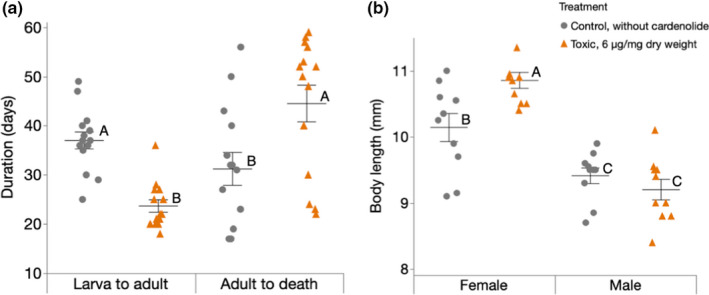
Effect of cardenolides on developmental time, lifespan, and body size of *Oncopeltus fasciatus*. (a) Each horizontal line represents the mean (± SE) number of days taken by *O*. *fasciatus* larvae (*n* = 14 per treatment) to turn adult (left side), and until death after reaching adulthood (right side, n = 14 per treatment); within the same category (left or right), different letters indicate significant differences between diets. (b) Each horizontal line represents the mean (± SE) body length of adult (female or male) *O*. *fasciatus* (*n* = 10 per treatment); different letters above bars indicate significant differences. Larvae were either raised on toxic diet or a control diet. Dots represent jittered raw data

Dietary cardenolides showed substantial effects on the developmental speed and longevity of *O*. *fasciatus*. Larvae raised on cardenolide‐containing diet developed faster into adults [*F*(1, 26) = 42.79, *p* < .001, LSMeans differences Student's *t*‐test] and adults resulting from larvae raised on cardenolide‐containing diet lived longer [*F*(1, 26) = 6.39, *p* = .02, LSMeans differences Student's *t* test] compared to individuals raised on cardenolide‐free diet (Figure 4a). Furthermore, cardenolides affected adult body size [*F*(3, 36) = 22.31, *p* < .001]. Females raised on toxic diet were the largest across all combinations (i.e., sex vs. diet; *p* < .001, when compared to males on toxic or control diets; and *p* = .02, when compared to females on the control diet, LSMeans Tukey HSD). The body length of male *O*. *fasciatus* was not different between the two diets (*p* = .74, LSMeans Tukey HSD) (Figure 4b).

### 3.4 Influence of dietary cardenolides on reproductive fitness of *Oncopeltus fasciatus*

**FIGURE 5:**
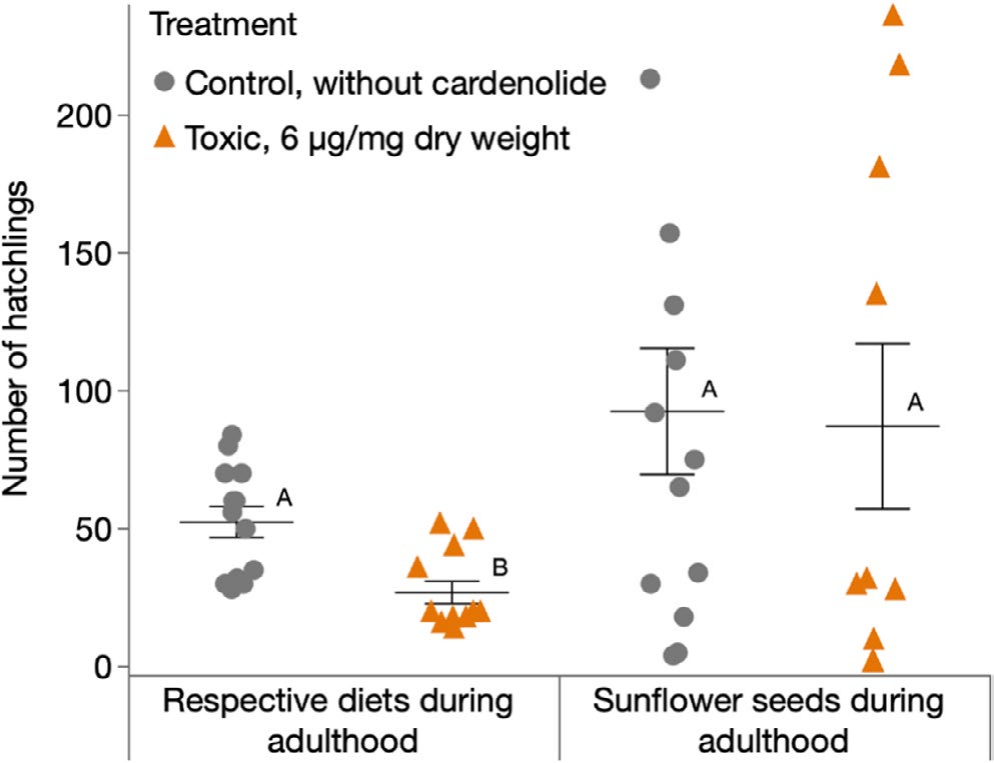
Reproductive success of *Oncopeltus fasciatus* in the presence or absence of dietary cardenolides. Each horizontal bar represents the mean (±SE) total number of hatchlings produced by *O*. *fasciatus* females until death. Larvae were either raised on toxic diet or control diet until adulthood. A group of bugs stayed on the respective diets after reaching adulthood (left; *n* for control = 13, *n* for toxic = 12), while the other group was transferred to sunflower seeds (right; *n* = 13 for control, *n* = 10 for toxic). Females producing no viable eggs at all were excluded from the analysis. Within the same group, different letters indicate significant differences. Dots and triangles represent jittered raw data

We observed substantial effects on the reproductive fitness of female *O*. *fasciatus* upon exposure to dietary cardenolides. When we continued feeding females after reaching adulthood on the same diet they were fed upon as larvae, *O*. *fasciatus* on cardenolide‐containing diets produced less hatchlings than individuals raised on cardenolide‐free diet [*F*(1, 23) = 15.82, *p* = .001, LSMeans differences Student's *t*‐test]. In contrast, *O*. *fasciatus* from cardenolide‐containing and cardenolide‐free diets produced similar numbers of hatchlings, when both groups were fed with sunflower seeds after reaching adulthood [*F*(1, 21) = 0.52, *p* = .48, LSMeans differences Student's *t*‐test] (Figure 5).

## 4 DISCUSSION

We investigated if dietary cardenolides affected the growth of closely related species of milkweed bugs (*O*. *fasciatus*, *C*. *nerii*, *S pandurus*, and *A*. *longiceps*) and the outgroup species, *P*. *apterus* possessing different combinations of the traits “cardenolide resistance” and “cardenolide sequestration” and having different dietary strategies (generalist vs. specialist). Remarkably, dietary cardenolides increased growth in the sequestering specialists, *O*. *fasciatus* and *C*. *nerii*, but not in the sequestering generalist *S*. *pandurus*. *Oncopeltus fasciatus* nymphs completed their development 2 weeks earlier and lived on average 13 days longer during the adult stage under cardenolide exposure when compared to individuals raised on control diet, but produced less offspring unless being transferred to a cardenolide‐free diet (sunflower seeds) after reaching adulthood.

Empirical evidence on the effect of dietary plant toxins on insect growth is ambiguous as different studies suggest contradictory effects. Nevertheless, several studies found effects that are in agreement with our study. For example, the growth of *O*. *fasciatus* was faster when raised on *Asclepias* species containing higher amounts of cardenolides (*A*. *syriaca* and *A*. *hirtella*) than when raised on species with lower cardenolide contents (*A*. *incarnata* and *A*. *viridiflora*) (Chaplin & Chaplin, 1981). Additionally, the African danaid butterfly *Danaus chrysippus*, having a cardenolide‐resistant Na^+^/K^+^‐ATPase (Petschenka et al., 2013), developed faster and produced larger adults when reared on *Calotropis procera* containing cardenolides compared to caterpillars raised on *Tylophora* spp. lacking cardenolides (Rothschild et al., 1975). Furthermore, *Zygaena filipendulae* larvae reared on the plant *Lotus corniculatus* containing cyanogenic glucosides developed faster, and the larvae showed decelerated development when reared on transgenic *L*. *corniculatus* free of cyanogenic glucosides (Zagrobelny et al., 2007).

In contrast to these studies, caterpillar growth of the milkweed butterfly species *Euploe core*, *D*. *plexippus*, and *D*. *gilippus* was unaffected by cardenolides across eight *Asclepias* species ranging from very low to very high cardenolide contents (Petschenka & Agrawal, 2015). Notably, all the studies mentioned above focused on insect feeding performance on intact plants or plant organs such as leaves or seeds that naturally represent highly complex diets (but see Bowers, 1984). This could be one reason why contradicting outcomes in response to the same class of chemical compounds were observed even within related plant species. Here, we used an artificial diet to control for variation across dietary treatments rigorously.

We showed that cardenolides had a positive effect on growth in *O*. *fasciatus* and *C*. *nerii*, both of which may be categorized as dietary specialists feeding on seeds of *Asclepias* spp. and seeds of *Nerium oleander*, respectively. We speculate that the positive impact on growth upon exposure of toxins may be due to selection on the resistant Na^+^/K^+^‐ATPases to function optimally in a “toxic environment,” that is, in the body tissues of a milkweed bug storing large amounts of cardenolides. In other words, there could be functional trade‐offs of cardenolide‐adapted Na^+^/K^+^‐ATPases in a physiological environment that is devoid of cardenolides, a phenomenon that could be called “evolutionary addiction.” Alternatively, better growth could be due to increased consumption of the larvae mediated by cardenolides as phagostimulants (Pantle & Feir, 1976). Nevertheless, our excretion data hint toward equal consumption of diet regardless of dietary cardenolide concentration (Figure S4). Since endogenous and sequestered defenses may trade‐off (Engler‐Chaouat & Gilbert, 2007), the lack of cardenolides for sequestration could lead to a higher investment towards endogenous defenses (i.e., defensive secretions) and therefore impair growth on the cardenolide‐free control diet. Although such a trade‐off has been suggested to occur in the milkweed bug *Lygaeus equestris* (Havlikova et al., 2020), metathoracic scent glands were shown to be reduced in *O*. *fasciatus* and other milkweed bugs (Aldrich, 1988; Schaefer, 1972) making this explanation rather unlikely.

The milkweed bug species *A*. *longiceps* is specialized on plants producing no cardenolides such as *Platanus* spp. or *Ulmus* spp. However, due to its evolutionary history, *A*. *longiceps* possesses resistant Na^+^/K^+^‐ATPases, but has lost the ability to sequester cardenolides (Bramer et al., 2015). In contrast, *S*. *pandurus* possesses resistant Na^+^/K^+^‐ATPases, and feeds on a wide array of host plants (Péricart, 1998; Vivas, 2012), including cardenolide producing species such as *Nerium oleander* and species of *Calotropis* from which it sequesters cardenolides (Abushama & Ahmed, 1976; Von Euw et al., 1971). For both species, dietary cardenolides did not influence growth, that is, they grew equally well on all diets.

The lack of a positive effect of dietary cardenolides on growth in *A*. *longiceps* may be associated with the inability of this species to sequester cardenolides. Accordingly, its Na^+^/K^+^‐ATPases may have undergone a different selection regime compared to the sequestering species. In other words, their putative suite of Na^+^/K^+^‐ATPases may have adapted to the absence of cardenolides in the body tissues secondarily. Alternatively, or in addition, there could be further physiological mechanisms involved mediating between sequestration and growth such as gaining energy from metabolizing sequestered toxins. Nevertheless, the latter seems rather unlikely as an explanation for increased growth in *O*. *fasciatus* and *C*. *nerii*, since the sugar moieties in digitoxin are dideoxy sugars not known to be easily metabolized as an energy resource (Liu & Thorson, 1994) and ouabain (carrying a rhamnose moiety) was found to be sequestered as such (Scudder & Meredith, 1982).

For *O*. *fasciatus*, we found negative correlations between sequestered cardenolides and body dry masses within the dietary treatments, which contrasts with the observed increased growth under cardenolide exposure. This correlation, however, was weaker on the low and the medium diet compared to the diet with the highest concentration of cardenolides. At the same time, growth in *O*. *fasciatus* was highest on low and medium diet compared to the most toxic diet which could point to a dose dependency of the observed positive effect of sequestered cardenolides, suggesting that our findings are not necessarily in contrast with the increased growth under cardenolide exposure. In addition, we used fresh weights for the growth experiments and dry weights for the assessment of sequestration which may further complicate the comparison of these two experiments.

While it is not surprising that cardenolide‐resistant Na^+^/K^+^‐ATPases alleviate toxicity of dietary cardenolides in both species, *A*. *longiceps* and *S*. *pandurus*, it is an open question why the sequestering *S*. *pandurus* did not show increased growth under cardenolide exposure. Although *O*. *fasciatus* sequesters substantially higher amount of cardenolides in comparison to *S*. *pandurus*, it seems unlikely that the extent of sequestration is the underlying mechanism, given that *C*. *nerii* sequesters concentrations similar to *S*. *pandurus* not showing increased growth. Along the same lines, cardenolide metabolism may also not explain the patterns of growth since we found pronounced differences in metabolism between *O*. *fasciatus* and *C*. *nerii* both showing increased growth under cardenolide exposure. However, the putative adaptations underlying generalist feeding behavior of *S*. *pandurus* likely caused different selection pressures in this species and may thus interfere with the pattern of amino acid substitutions across the different Na^+^/K^+^‐ATPases or lead to differential expression of the Na^+^/K^+^‐ATPase genes across different tissues.

The outgroup species, *P*. *apterus*, belongs to a different family (Pyrrhocoridae) and is not adapted to cardenolides, that is, it has a cardenolide‐sensitive Na^+^/K^+^‐ATPase and is not able to sequester cardenolides (Bramer et al., 2015). The lack of a resistant Na^+^/K^+^‐ATPase most likely explains why larval growth in this species was compromised substantially by dietary cardenolides. Alternatively, or in addition, reduced growth could be due to feeding deterrence mediated by dietary cardenolides as indicated by the reduced amount of excretion products observed during our feeding trial.

Although specialist insects can successfully feed on toxic host plants, it is generally expected that the underlying resistance traits incur costs via trade‐offs, which are expected to depend on the ecological context and the molecular mechanisms involved (Peterson et al., 2016; Smilanich et al., 2016). As *O*. *fasciatus* raised on cardenolide‐containing diet developed faster into adults and had a longer lifespan compared to those raised on cardenolide‐free diet, cardenolide exposure clearly influences the fecundity‐longevity trade‐off. A faster development and an extended lifespan both indicate higher fitness. Nevertheless, individuals exposed to dietary cardenolides produced a lower number of hatchlings contradicting higher fitness. This apparent disadvantage, however, could be alleviated by maternal transfer of cardenolides to the eggs mediating protection against predators (Newcombe et al., 2013). Moreover, we found that feeding on a non‐toxic diet (i.e., sunflower seeds) as adults can compensate for this effect. This likely resembles the natural situation since *O*. *fasciatus* larvae feed and cluster around on *Asclepias* seeds or seedpods, while adults disperse and forage on various plants (Feir, 1974). Contrary to our findings, it was shown that male *O*. *fasciatus* fed with *A*. *syriaca* seeds invested in reproduction at the expense of survival when compared to those fed with sunflower seeds (Attisano et al., 2012). In this study, however, only the diet of male specimens was manipulated and female *O*. *fasciatus* were exclusively fed with sunflower seeds. Moreover, due to the use of milkweed seeds, it is not possible to directly attribute the observed effects to cardenolides. In conclusion, it seems likely that cardenolides exert a positive effect on overall fitness in *O*. *fasciatus*, which is in disagreement with theory predicting costs of sequestration.

Dealing with xenobiotics can require energy allocation towards metabolism or can cause pleiotropic effects due to particular molecular mutations that might confer a selective advantage in the presence of xenobiotics but incur a cost in their absence (Coustau et al., 2000; Mauro & Ghalambor, 2020). Although it is unclear how the function of Na^+^/K^+^‐ATPase could be related to growth and longevity in milkweed bugs, the enzyme is involved in many physiological processes apart from being a cation carrier suggesting many unknown non‐canonical functions of Na^+^/K^+^‐ATPase (Liang et al., 2007), which could provide a mechanistic link. In *O*. *fasciatus*, the three gene copies (α1A, α1B, and α1C) encoding α‐ subunit of cardenolide‐resistant Na^+^/K^+^‐ATPases have diverse functions (Lohr et al., 2017) and duplicated α1 gene copies do not only vary in number and identity but also show specific expression patterns in different body tissues (Dobler et al., 2019; Yang et al., 2019; Zhen et al., 2012) allowing for complex regulation.

In conclusion, reduced growth in the absence of cardenolides in highly specialized milkweed bugs with cardenolide‐resistant Na^+^/K^+^‐ATPases suggests a novel type of physiological cost arising in the absence of plant toxins. Mechanistically, this cost could be due to negative pleiotropic effects mediated by resistant Na^+^/K^+^‐ATPases not functioning optimally in a physiological environment lacking cardenolides. Furthermore, the observed effects of cardenolides on the fecundity‐longevity trade‐off probably leading to increased fitness in *O*. *fasciatus* may be due to optimized resource allocation under the influence of sequestered cardenolides. Our study suggests a concept that is contradictory to the general assumption that sequestered plant toxins produce physiological costs and our results indicate a further level of coevolutionary escalation.

## CONFLICT OF INTEREST

The authors declare no conflict of interest.

## AUTHOR CONTRIBUTIONS

**Prayan Pokharel:** Conceptualization (equal); data curation (lead); formal analysis (equal); investigation (lead); methodology (equal); validation (equal); visualization (lead); writing – original draft (equal); writing – review and editing (equal). **Anke Steppuhn:** Formal analysis (equal); supervision (supporting); validation (equal); writing – review and editing (equal). **Georg Petschenka:** Conceptualization (lead); data curation (equal); formal analysis (equal); funding acquisition (lead); methodology (equal); project administration (lead); resources (lead); supervision (lead); validation (equal); writing – original draft (equal); writing – review and editing (equal).

## Supporting information

Supplementary Material

## Data Availability

All raw data reported in this manuscript were archived in Dryad: https://doi.org/10.5061/dryad.q2bvq83m3.
