# Supplementary material for: Dietary cardenolides enhance growth and change the direction of the fecundity‐longevity trade‐off in milkweed bugs (Heteroptera: Lygaeinae)
Source: Ecol Evol. 2021 Nov 30;11(24):18042–54. doi: 10.1002/ece3.8402 (PMC8717354; doi:10.1002/ece3.8402)
Supplement: Supplementary file 1 — Supplementary Material [file ECE3-11-18042-s001.docx]

# **Supplementary Information**

## **Supplementary Methods**

### **Quantification of excretion products**

We estimated the amount of food uptake by quantifying the amount of excretion products during our feeding assay. Specifically, the area of faecal stains on filter papers lining the Petri-dishes was analysed. We only analysed filter papers from Petri-dishes in which all three bugs survived until the end of the experiment (i.e. for three weeks). Filter papers were scanned and the stained area was quantified by following the instruction of image analysis (Reinking 2007) using ImageJ 1.52k (National Institutes of Health, US). Excretion data were log_10_-transformed to achieve homogeneity of variances and normality of residuals. To test for differences in excretion across treatments data were analysed by ANOVA followed by the LSMeans Tukey HSD test in JMP.

## **Supplementary Results**

### **Estimation of excretion area**

We estimated the area of excretion products on filter paper to assess bug feeding activity during our feeding trials. In line with increased growth of *P. apterus* on the control diet [F(3, 15) = 4.69, P = 0.02], we observed statistical differences of excretion products. Specifically, bugs excreted less waste products when fed on medium (P = 0.01) and high-dose (P = 0.04) but not on low dose diet (P = 0.22) compared to the control. In contrast, we found no statistical differences of excreted waste products across treatments in *C. nerii* [F(3, 14) = 1.38, P = 0.29], *S. pandurus* [F(3, 15) = 0.53, P = 0.67], and *A. longiceps* [F(3, 5) = 0.96, P = 0.48] (Supplementary Figure 4). *O. fasciatus* [F(3, 19) = 8.34, P < 0.001] excreted similar amounts when fed on either control, low or medium-dose diet. Interestingly, *O. fasciatus* excreted less in the high-dose (but similar to control) than compared to low and medium-dose. Moreover, there was a difference between excretion on medium (P = 0.002) and low dose (P = 0.004) diet as compared to the high dose diet.

# **Supplementary figures and legends**


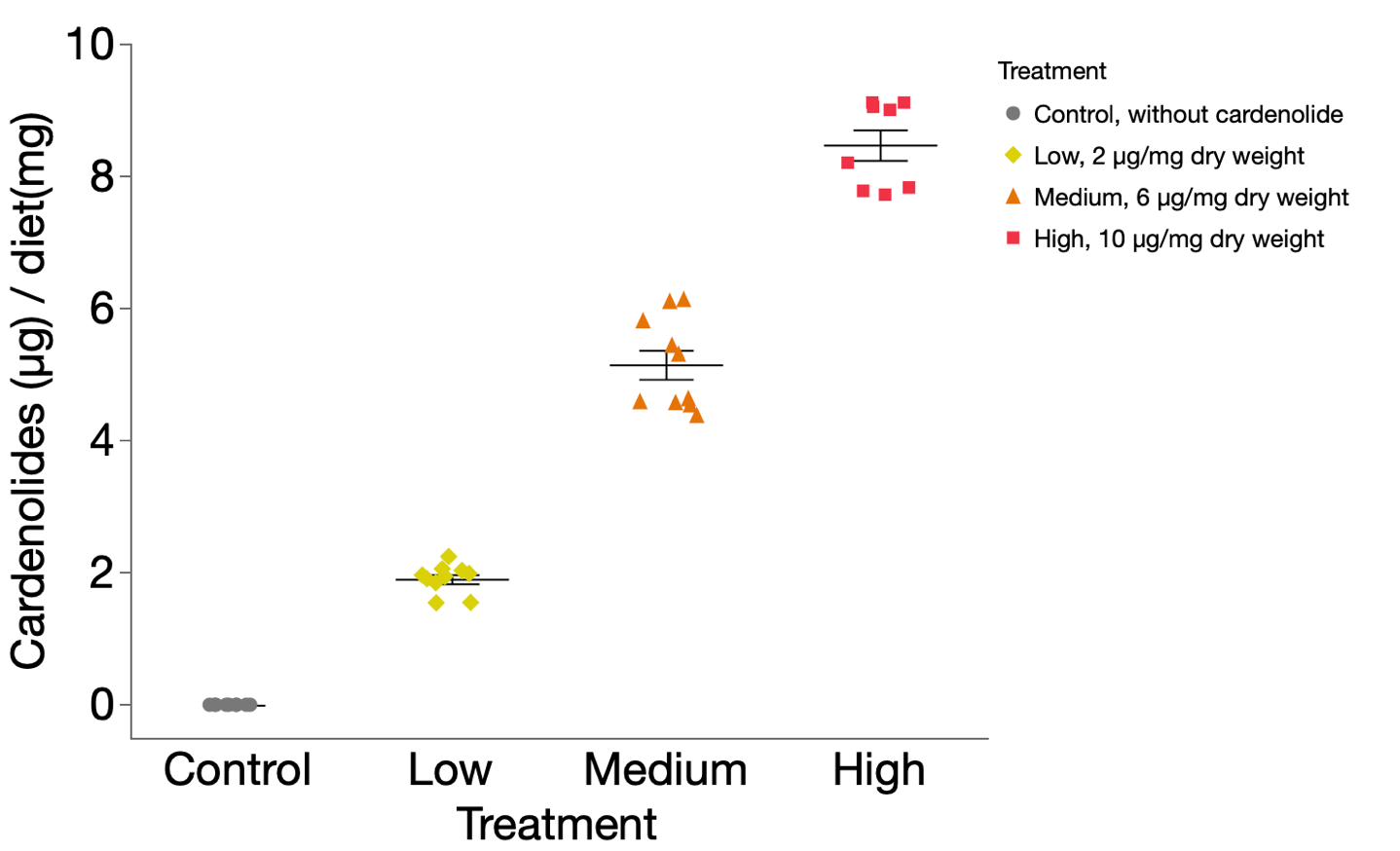


**Supplementary Figure 1** Concentration of cardenolides in the artificial diet. Each horizontal bar represents the mean concentration of cardenolides (± SE) in the diet (n = 10 per treatment). Symbols represent jittered raw data.


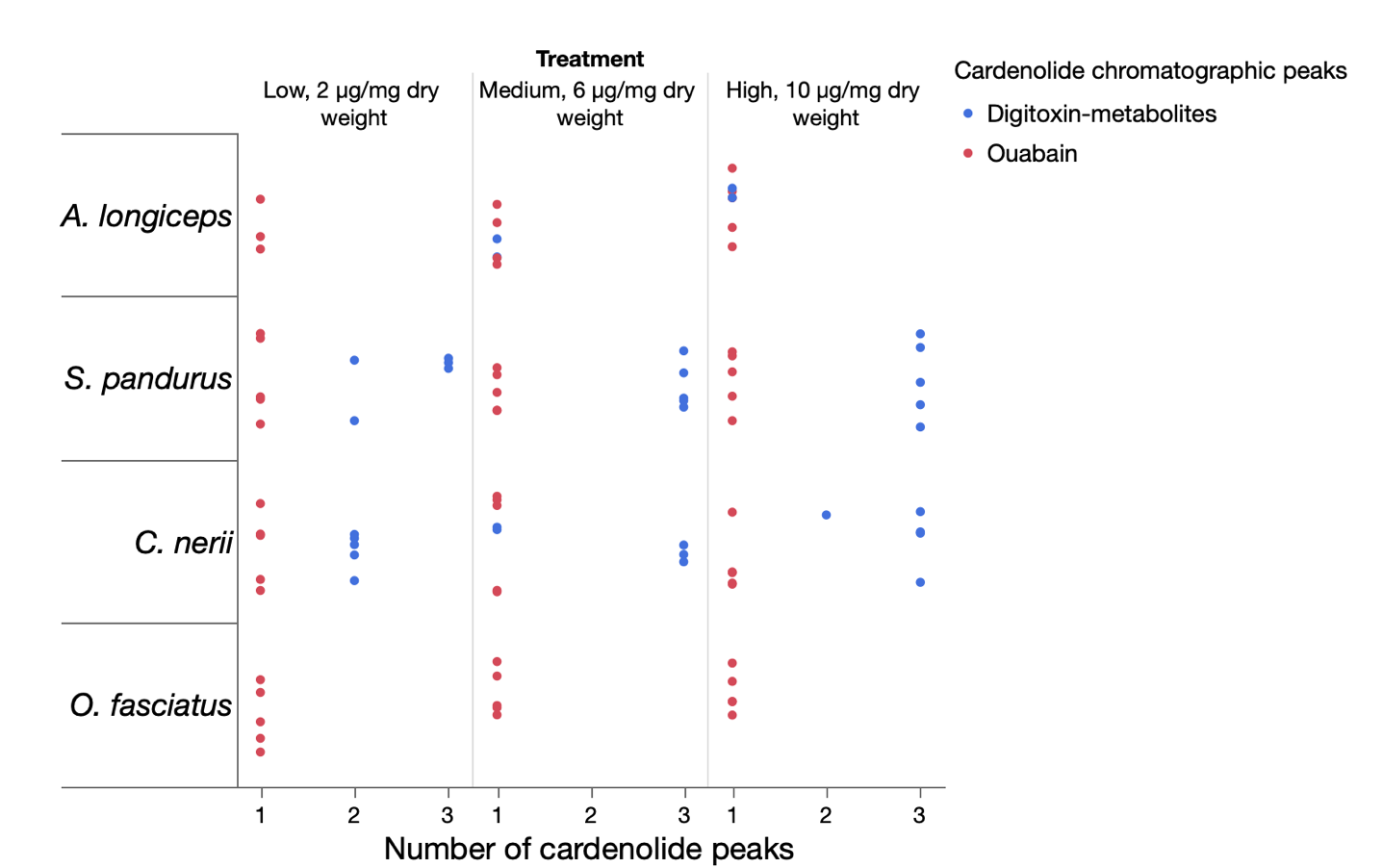


**Supplementary Figure 2** Structural diversity of sequestered cardenolide peaks by milkweed bugs. Each data point represents a bug specimen and the number of cardenolide peaks found in specimens of O. fasciatus (n = 10 per treatment), C. nerii (n = 5 per treatment), S. pandurus (n = 5 per treatment), and A. longiceps (n = 5 per treatment) when raised on artificial diet containing an equimolar mixture of ouabain and digitoxin. Chromatographic peaks with a cardenolide spectrum and a similar retention time like digitoxin were classified as digitoxin metabolites. Although we used a different HPLC method for O. fasciatus, the outcome is probably the same as if we had used the HPLC method used for C. nerii, S. pandurus, and A. longiceps. This assumption was validated by comparisons with O. fasciatus samples analysed during a different set of experiments (Heyworth et al., manuscript in preparation), hence it is valid to compare O. fasciatus to other species.

**
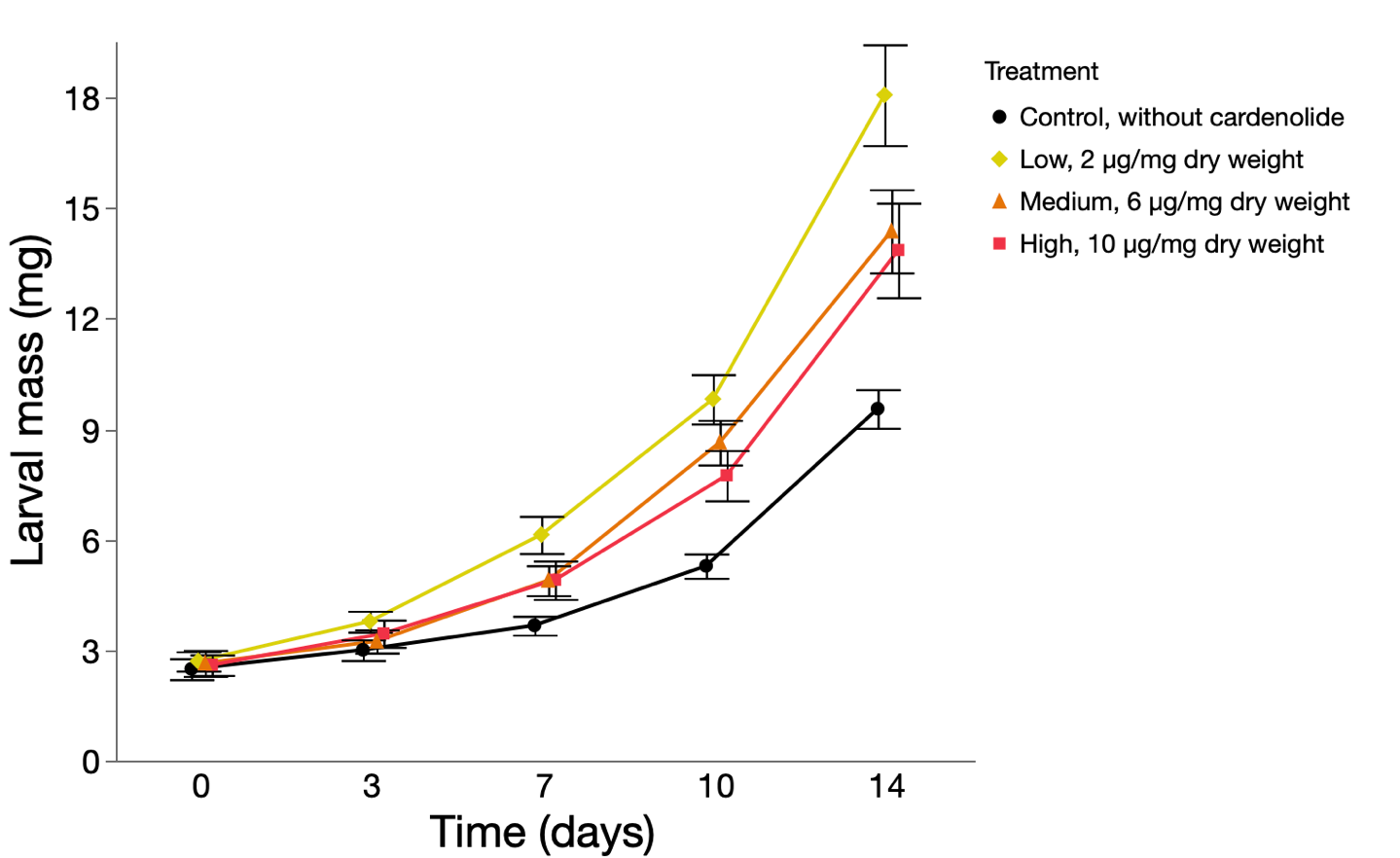
**

**Supplementary Figure 3** Growth of O. fasciatus on artificial diet with increasing doses of cardenolides using a genetically distinct O. fasciatus lab strain. Each data point represents the mean mass (± SE) of larvae raised on an equimolar mixture of ouabain and digitoxin (n = 10-12 per treatment). We commercially obtained O. fasciatus eggs from Carolina Biological Supply Company (Burlington, NC, US). Larvae were maintained on sunflower seeds as described above and used only in this feeding assay. Overall, cardenolides had a positive effect on growth [F(3, 39) = 5.53, P = 0.003, Repeated Measures ANOVA], but only low-dose (P = 0.002) was statistically significant from control, and not the medium (P = 0.07) and high-dose (P = 0.13, LSMeans Tukey HSD).


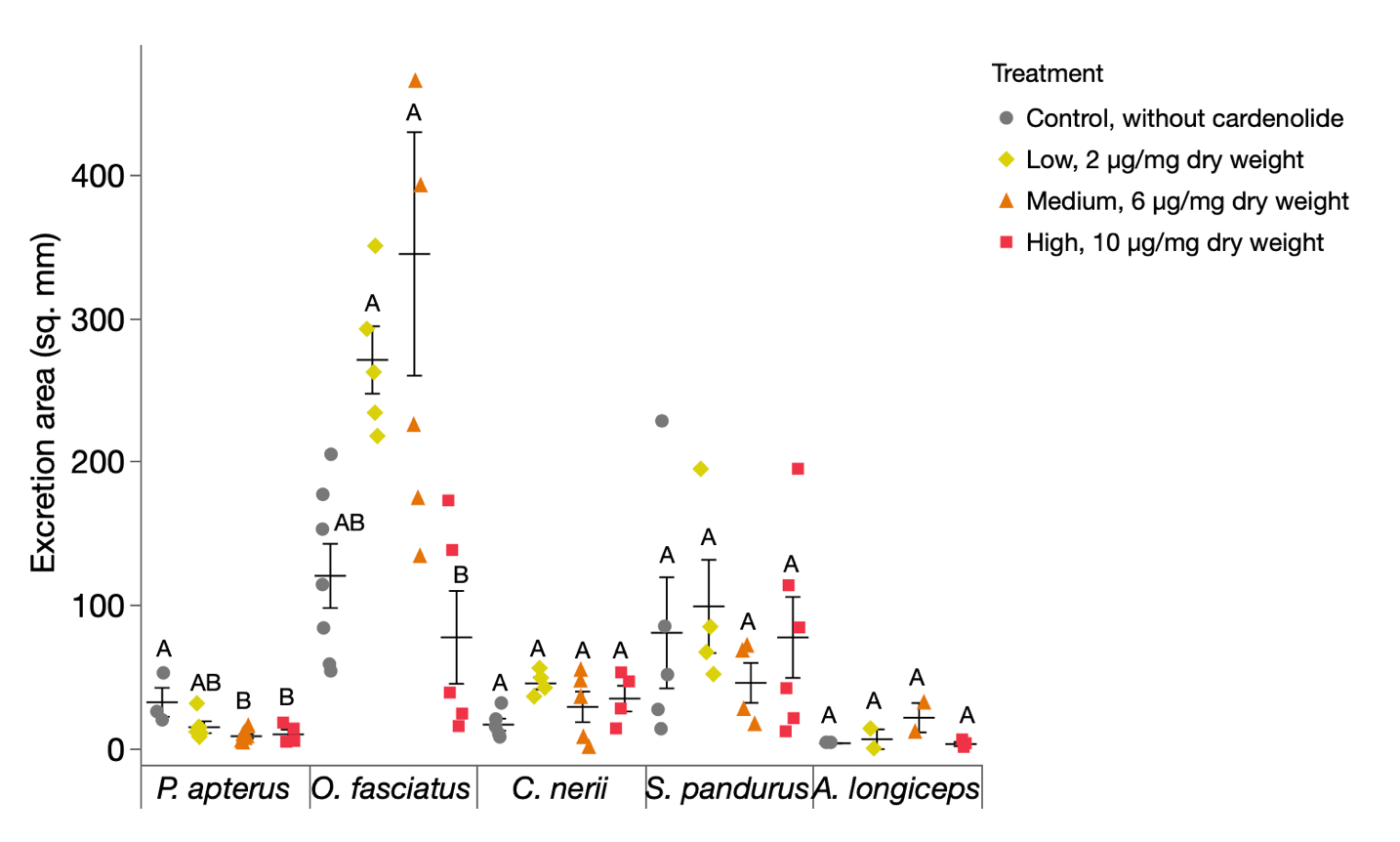


**Supplementary Figure 4** Amount of excretion products by bug species raised on increasing doses of cardenolides. Each horizontal bar represents the mean (± SE) of faecal stains on filter paper produced by P. apterus (n = 4-7 per treatment), O. fasciatus (n = 5-7 per treatment), C. nerii (n = 4-5 per treatment), S. pandurus (n = 5-6 per treatment), and A. longiceps (n = 2-3 per treatment) when raised on an equimolar mixture of ouabain and digitoxin. Within the same bug species, different letters indicate significant differences. Symbols represent jittered raw data.

**Supplementary Reference**

Reinking, L. (2007). Examples of image analysis using ImageJ. Department of Biology, Millersville University. Retrieved from https://imagej.nih.gov/ij/docs/pdfs/examples.pdf
